# Supplementary material for: The pluripotency factor NANOG controls primitive hematopoiesis and directly regulates Tal1
Source: EMBO J. 2019 Feb 27;38(7):e99122. doi: 10.15252/embj.201899122 (PMC6443201; doi:10.15252/embj.201899122)
Supplement: Supplementary file 1 — Expanded View Figures PDF [file EMBJ-38-e99122-s001.pdf]

Expanded View Figures

Figure EV1. Developmental phenotype of sustained *Nanog* expression in the mouse embryo.

A CD31 staining of yolk sac vasculature in control (−dox) or treated (+dox) E9.5 $Nanog^{tg}$ embryos. Below, higher magnifications of the boxed areas are shown. Scale bar, 500 μm.

B Heart morphology is not affected in dox-treated (+dox) E9.5 $Nanog^{tg}$ embryos. Below, hematoxylin eosin staining of sections reveal normal development of the heart in treated (+dox). Dotted lines in upper panels indicate plane of sections. Scale bar, 500 μm (whole mounts), 250 μm (sections).

C Representative images of May-Grünwald-Giemsa stained cytospins from control (−dox) and dox-treated (+dox) E9.5 embryos. Scale bar, 5 μm.

D Relative expression of *Nanog* and hematopoietic genes in $cKit^{+}CD41^{+}$ and $cKit^{-}CD41^{+}$ populations sorted from E9.5 control (−dox) and treated (+dox) embryos. $n = 7$ (−dox) or $n = 4$ (+dox); each replicate contained a pool of 5 (−dox) or 8 (+dox) E9.5 $Nanog^{tg}$ embryos. ***$P < 0.0005$; Student's *t*-test. Horizontal line represents mean values and error bars SD.

E Whole-mount *in situ* hybridization for *Gata1* and *Klf1* of control (−dox) and treated (+dox) E7.5 $Nanog^{tg}$ embryos. Arrows indicate the location of blood islands in the extraembryonic yolk sac. Scale bar, 250 μm.

F Relative expression of *Nanog*, mesodermal (*Eomes*, *Brachyury*, *Kdr*), and hematopoietic (*Runx1*, *Tal1*, *Gata1*, *Klf1*) genes in single control (−dox) or treated (+dox) E7.5 embryos ($n = 4$). *$P < 0.05$, **$P < 0.005$, ***$P < 0.0005$; Student's *t*-test. Horizontal line represents mean values and error bars SD.

 © 2019 The Authors

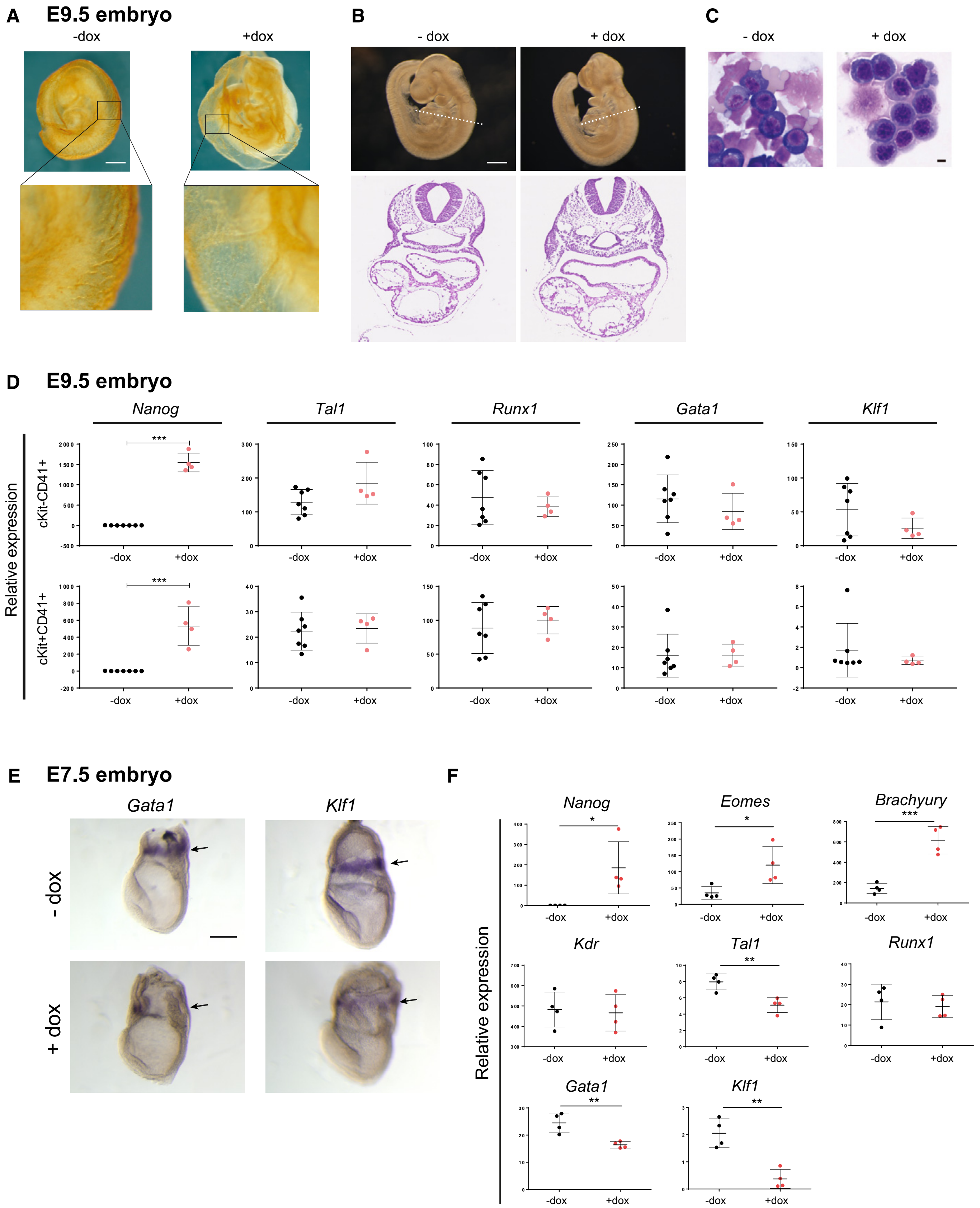
A
E9.5 embryo
-dox
+dox
B
- dox
+ dox
C
- dox
+ dox
D
E9.5 embryo
Nanog
Tal1
Runx1
Gata1
Klf1
Relative expression
cKit-CD41+
cKit+CD41+
-dox
+dox
***
E
E7.5 embryo
Gata1
Klf1
- dox
+ dox
F
Nanog
Eomes
Brachyury
Kdr
Tal1
Runx1
Gata1
Klf1
Relative expression
*
**
***

Figure EV1.

Figure EV2. Expression profiles of hematopoietic genes during differentiation aligned at the peak of *Brachyury* expression.

A Timing of *Brachyury* expression before (left) and after (right) the alignment of its peak of expression that occurs at day 3 (D3) of differentiation in wild-type ES cells (wt, black) and at day 5 (D5) in $Nanog^{-/-}$ cells (red). $n = 3$. Horizontal line represents mean values and error bars SEM.

B Timing of expression of *Nanog* and selected hematopoietic genes when wt and $Nanog^{-/-}$ cells after alignment. The time point of maximum *Brachyury* expression is labeled as T d0. Horizontal line represents mean values and error bars SEM.

C Relative expression of *Nanog*, *Brachyury*, *Tal1*, *Gata1*, *Klf1*, and *Hbb-bh1* determined by RT–qPCR for $Nanog^{flox/-}$ and $Nanog^{del/-}$ during ES to EpiL cell transition ($n = 3$). $^*P < 0.05$, $^{**}P < 0.01$, $^{***}P < 0.001$, $^{****}P < 0.0001$; ANOVA with Fisher post-test. Horizontal line represents mean values and error bars SEM.

 © 2019 The Authors

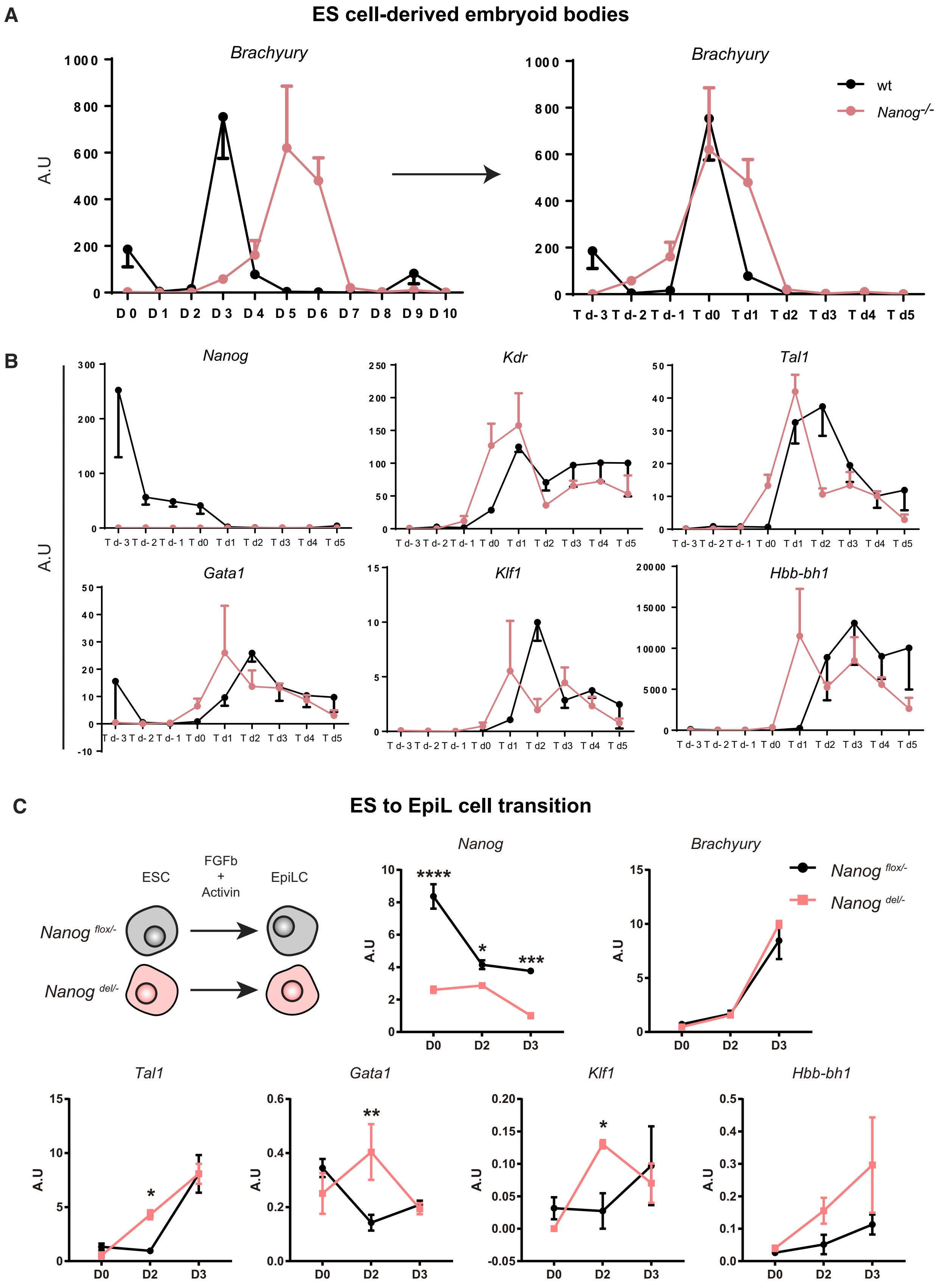
A
ES cell-derived embryoid bodies
Brachyury
Brachyury
wt
Nanog-/-
A.U
D 0 D 1 D 2 D 3 D 4 D 5 D 6 D 7 D 8 D 9 D 10
T d-3 T d-2 T d-1 T d0 T d1 T d2 T d3 T d4 T d5
B
Nanog
Kdr
Tal1
Gata1
Klf1
Hbb-bh1
C
ES to EpiL cell transition
ESC
FGFb + Activin
EpiLC
Nanog flox/-
Nanog del/-
Nanog
Brachyury
Tal1
Gata1
Klf1
Hbb-bh1
D0 D2 D3

Figure EV2.

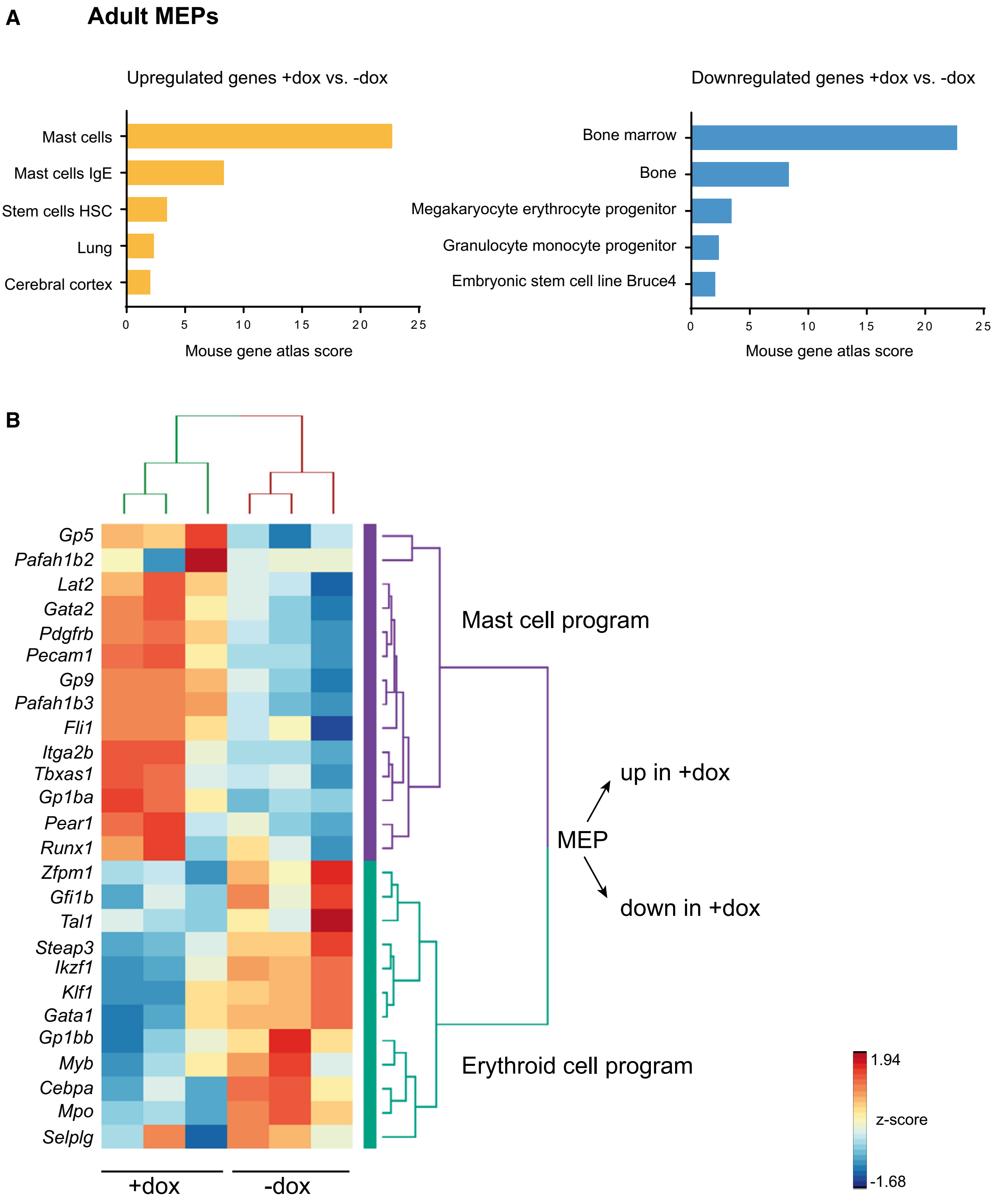

Figure EV3. Transcriptional profiling of MEPs from *Nanog*^{tg} adult mice.

A Enriched functional categories in genes that are significantly upregulated (left, orange) or downregulated (right, blue) in MEPs isolated from dox-treated *Nanog*^{tg} mice compared to untreated controls. Mouse gene atlas score was calculated using Enrichr.

B Heatmap of the expression (as z-score) across the three replicates for each condition of selected genes for the mast cell and erythroid transcriptional programs.

© 2019 The Authors

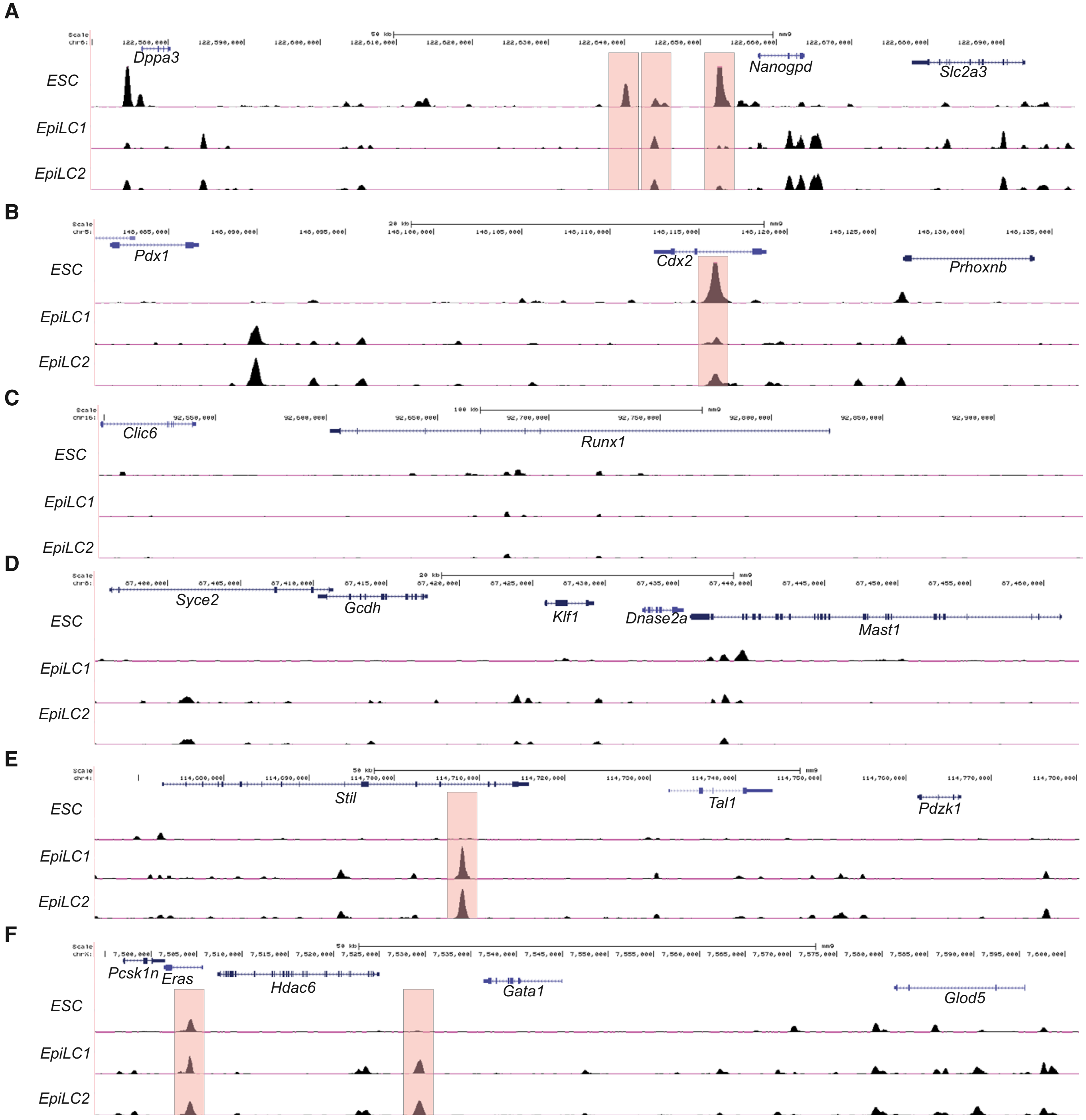

Figure EV4. Distribution of NANOG bound regions at selected loci in ES and Epi-like cells (EpiLC).

A–F UCSC browser views (mm9) of different genomic regions showing NANOG bound regions as determined by ChIP-seq in ES cells (*ESC*) and two replicates of epiblast-like cells (*EpiLC1*, *EpiLC2*). Selected peaks are highlighted by boxing in red. (A) *Nanog*: chr6:122,569,855-122,699,399. (B) *Cdx2*: chr5:148,080,850-148,136,544. (C) *Runx1*: chr16:92,497,828-92,940,224. (D) *Klf1*: chr8:87,395,041-87,462,459. (E) *Tal1*: chr4:114,664,868-114,780,341. (F) *Gata1*: chrX:7,493,906-7,601,563. ChIP-seq data were obtained from Murakami *et al*, 2016 (GEO accession number GSE71933).

© 2019 The Authors

A

mm9, chr4:114707745-114708744

```
ATGAGACCAGTGCAAACTGTATAGAACAACCCTGCCCTGAAAAACAAAACAAAACAAAAC
ATTTCCAGAGCTGTCCGTGGTGGCTTCGGTGCTGTTGCGCAAGCATCTAGTCTGACTCAT
GCTGTGGCTCTCATTGCTCAGTATTCATCGTGTCATTAGCTCCCTGGGCTCCCAGATCAG
CCAGTCGTTGTGCTGCTGGAGCAGTCTCTAAAGATGAGTTTATTGGCTGTACAGCTTATT
GGCTATCGATTGCTTTTTCCCAATGGAAAGTGATGGTACAAGGAGACATTGTTTAAAACA
ACTGTTTAAGAAGCCATGCCAAATTTGCAGGTAATGGCACATTGAAGTCATTGTTTAAAA
TTTTTTTAGGTTAAAATCTTTCGTATCTCTTCCAGCCTTTTTTTTATTTACTACTTTAGA
CACCTAGATAAATTTTTGAGCACAGTGTTGGAGCTCAGTGATTATGAGCACACTCATTGG
CTCAAGCTTCTGATTTTAGTCATGGCTATGAACTTTTCTCTGCTCCCTGGTTGACCTATC
AGATGGGAATAATAAAGGCACCTATTTTTGAGGTTGTTGTGAGAAGTAAAGGATCCTATG
TCATCAGTCAGAACAGTGCCTGCCACTGGGTAGCCAGCGTTTGTTTGGTGGTGAATACTC
TGAAAGGAGGAGATTATGTGGCTTTTGGTTTCAGTCCTAGCAGCTGGCTGCAGTGCTGTG
GGCTGTTGGTGATTCTGTCGCATATGGCAGGACACAGAACCTGAGCTCGGGAGTGGACTT
TCTTCACCCTCCCCCCCAGCGGATTAGACAATGCTGTCCACATTCCGGGCTGCTCTTCTC
TCAGTTGCTGTTCCTCTGTAAAATGCCCTCACAGACACACCTGCAGTGTGCTTTAGGAAT
CTCCCAGGCAAGACTACTCACCATCACAGTGTTTAATCACGATGGTCATTTAGTTAAAAA
TGGAATAAGCCACATTCAGTAACTGGCATGGACTCTCAGA
```

PCR genotyping primers

guide RNA

NANOG BS

B

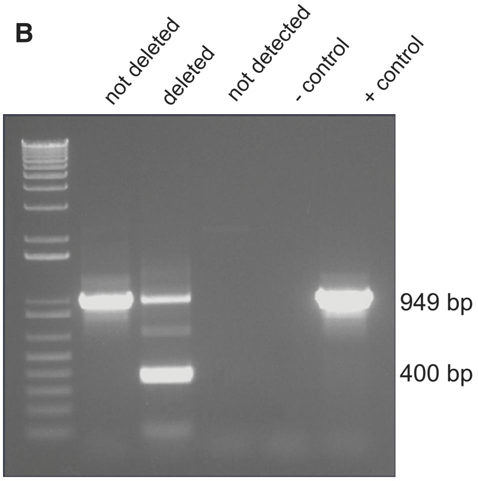

C

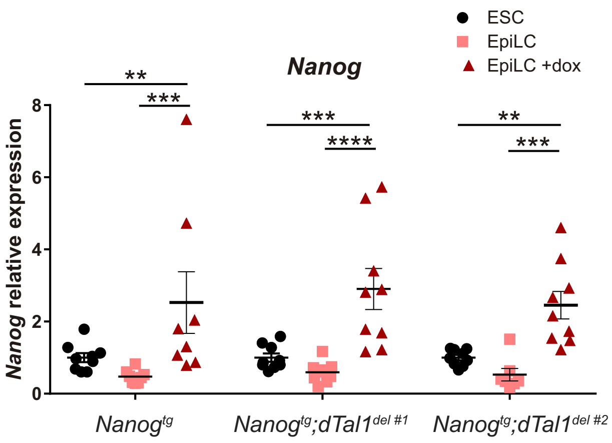

Figure EV5. CRISPR/Cas9 deletion of the *Tal1* NANOG binding peak.

A DNA sequence of the genomic region located at −22 kb from *Tal1* bound by NANOG in EpiLC. PCR genotyping primers are highlighted in yellow, guide RNAs in blue (PAM sequence is underlined), and two consensus NANOG binding motifs in dark gray and white bold lettering.

B Representative gel of PCR genotyping of individual E6.5 embryos showing not deleted, deleted, not detected, negative control (no DNA), and positive control (wild-type embryo). The size of the wild-type (949 bp) and deleted (400 bp) bands are indicated.

C Relative expression of *Nanog* determined by RT–qPCR for each ES cell line (ESC; n = 9 for all three lines) and EpiL cells without (EpiLC; EpiLC; $Nanog^{tg}$ and $Nanog^{tg}$; $dTal1^{del\#1}$, n = 8; $Nanog^{tg}$;$dTal1^{del\#2}$, n = 7) or with dox treatment (EpiLC +dox; $Nanog^{tg}$, n = 8; $Nanog^{tg}$;$dTal1^{del\#1}$ and $Nanog^{tg}$;$dTal1^{del\#2}$, n = 9). The genotype of the cell lines is indicated below. **P < 0.01, ***P < 0.001, ****P < 0.0001; ANOVA with Fisher post-test. Horizontal line represents mean values and error bars SEM.

© 2019 The Authors
